# Trace Elements in Different Blood Products Used in Neonatal Transfusion: Arsenic and Selenium

**DOI:** 10.3390/ijms26188853

**Published:** 2025-09-11

**Authors:** Sanaa M. Aly, Hidi A. A. Abdellatif, Yasmine G. Mohamed, Radwa A. M. Soliman, Mohamed Osama Abdalla, Nada Hosny Ahmed Ali, Abdullah A. Hashish, Nicolas Beauval, Jean-Michel Gaulier, Delphine Allorge, Nancy Shalaby, Ahmed Omran

**Affiliations:** 1 Forensic Medicine and Clinical Toxicology Department, Faculty of Medicine, Suez Canal University, Ismailia 41522, Egypt; 2 CHU Lille, Service de Toxicologie-Génopathies, F-59000 Lille, France; 3 Clinical Medical Sciences Department, Faculty of Medicine, King Salman International University, South Sinai 46618, Egypt; 4 Medical Biochemistry and Molecular Biology Department, Faculty of Medicine, Suez Canal University, Ismailia 41522, Egypt; haidy.azmy@med.suez.edu.eg; 5 Medical Biochemistry and Molecular Biology Department, Faculty of Medicine, King Salman International University, Tur Sinai, South Sinai 46618, Egypt; 6 Oncology Diagnostic Unit, Faculty of Medicine, Suez Canal University, Ismailia 41522, Egypt; 7 Department of Pediatrics and Neonatology, Faculty of Medicine, Suez Canal University, Ismailia 41522, Egypt; 8 Department of Clinical Pathology, Faculty of Medicine, Suez Canal University, Ismailia 41522, Egyptahsahish@ub.edu.sa (A.A.H.); 9 Pathology Department, College of Medicine, University of Bisha, Bisha 61922, Saudi Arabia; 10 Université de Lille, ULR 4483—IMPECS—IMPact de l’EnvironnementChimique sur la Santéhumaine, 59000 Lille, France; 11 Forensic Medicine and Clinical Toxicology Department, Faculty of Medicine, Damietta University, New Damietta 34517, Egypt

**Keywords:** blood product, trace element, blood transfusion, neonates

## Abstract

Arsenic (As) is a toxic trace element with neurodevelopmental, carcinogenic, and other adverse effects. Meanwhile, selenium (Se) is an antioxidant trace element with essential physiological roles in humans. The preterm neonate is the most heavily transfused patient. The multiple blood transfusions could expose this vulnerable group to trace elements with variable effects. This study aimed to quantify As and Se in various blood products that were used in neonatal blood transfusions alongside an estimate of a dose per transfusion. In addition to exposure quantification, database mining and molecular docking analysis were performed to explore potential detoxification strategies. Samples from transfusion bags: *N* = 120; 30 samples of each type of blood product (plasma, platelets, packed RBCs (pRBCs), and whole blood “WB”) were analyzed for As and Se by using Inductively Coupled Plasma Mass Spectrometry (ICP-MS). The As and Se medians of all blood units were 0.6 and 74 μg/L, respectively. About 20% of donors have As levels above 1 μg/L. In addition, 74% of donors have Se levels less than 100 μg/L (the level of sub-optimal activity of the antioxidant enzyme glutathione peroxidase), and 60% of the donors have Se levels below the accepted minimum Se level (80 μg/L). The pRBCs were the units with the highest As and Se content. Meanwhile, WBs were the units with the highest dose per transfusion. Key methyl donors—folic acid, S-adenosylmethionine (SAM), and glutathione (GSH)—showed strong binding affinity to the active site of arsenite methyltransferase (AS3MT), a crucial enzyme in As metabolism. These ligands interacted with conserved catalytic residues such as ASN173, ASP115, and CYS92, suggesting a supportive role in enhancing As methylation and clearance. The present study highlights that neonates are exposed to As and Se via different blood product transfusions with high potential to increase As and decrease Se after transfusion. It is recommended to select donors and screen blood units with optimal Se levels and minimal As content for neonatal transfusions. The integration of in silico docking with exposure assessment adds mechanistic insight and highlights the potential for targeted nutritional interventions to reduce As toxicity in vulnerable neonatal populations.

## 1. Introduction

Very low birth weight (VLBW) and critically ill neonates are categories of patients with more frequently administered transfusion therapy [1]. Different blood product transfusions are commonly used in various medical conditions (coagulation disorders, thrombocytopenia, severe anemia, and exchange/intrauterine transfusion) [2]. The VLBW and critically ill neonates are therefore susceptible to various elements that may be exposed due to transfusion. These elements are natural components that have different chemical characteristics and toxicological predilections [3]. They are often categorized as essential, probably essential, and potentially toxic trace elements (TEs). Essential TEs are required for vital roles in humans, such as Selenium (Se) and Zinc. Potentially essential TEs are thought unlikely to have a beneficial function in the life process of humans, such as manganese. The potentially toxic TEs have no known beneficial biological role, such as lead, mercury, cadmium (Cd), and arsenic (As) which are notable for their toxicity even at low levels of exposure [4]. Lead, mercury, and Cd are the most common toxic TEs [5,6] that were extensively studied before [7,8,9,10,11]. The negative impact of these potentially toxic TEs, including As, on human health is still being observed and studied [12]. Safe levels for intravenous administration of these TEs are unknown [13]. Other elements, such as Se, are essential TEs that are incorporated into enzymes and proteins and have antioxidant properties with crucial roles in the defense against oxidative stress [14]. Thus, it has been reported that Se could diminish As or Cd toxicity [12]. Although AS and Se are different types of TEs, the toxic effects of As and Se depend on their chemical forms [15].

Arsenic is an environmental contaminant with different forms. Arsenite (As^III^) and arsenate (As^V^) are the most toxic forms found in water. Arsenobetaine (AsBet), a more complex arsenic compound, was identified essentially in seafoods [16]. Arsenic has severe undesired impacts [17] on learning, memory, and cognitive deficiencies through altered neurotransmitter levels [18,19]. The mean intelligence quotient (IQ) score of infants unexposed to As was 6 points higher than that of exposed infants [20]. The As concentrations were higher in children with autistic spectrum disorders than in control children [21]. It accumulates in different brain areas after crossing the blood–brain barrier [22] which causes As-induced neurotoxicity mainly through increased oxidative stress [23]. The International Agency for Research on Cancer (IARC), which belongs to the World Health Organization (WHO), classifies As and inorganic As compounds (including As^III^ and As^V^) as carcinogenic to humans. While the organic arsenic compounds: dimethylarsinic acid (DMA) and monomethylarsonic acid (MMA) are classified as possibly carcinogenic to humans [24].

Selenium is an essential micronutrient for humans that is mainly obtained through diet and/or nutritional supplementation [25]. It exists as organic Se compounds (selenate and selenite) that are derived mainly from animal foods. Selenocysteine is another organic form that is formed after ingestion of selenate and selenite, which are more bioavailable and less toxic than inorganic forms [26]. Inorganic Se is present in plants and water. Generally, the synthesis of selenoprotein is performed in the liver after intestinal absorption, and then it enters the bloodstream to supply Se to the other organs. The biological effects of Se are primarily mediated by selenoproteins [27]. Trace Se amounts are essential for maintaining optimal health, as Se is a component of the selenoproteins that participate in a wide range of cellular physiological processes such as redox homeostasis, inflammatory and immunological responses, carbohydrate metabolism, cardiovascular and reproductive health, and brain function maintenance [25]. It plays a role in the antioxidant system mainly by neutralizing the deleterious effects of free radicals and maintaining cell integrity [28,29]. It prevents morbidities in extremely preterm infants [30]. This group is at increased risk of developing Se deficiency because of the immature chorionic villi necessary for the effective transport of Se. A shortened gestational period leads to limited hepatic Se storage and compromised intestinal absorption of Se in the early neonatal period [31]. Se deficiency in this category was linked to increased late-onset sepsis, respiratory morbidity, chronic lung disease, retinopathy of prematurity, hemolytic anemia, sick euthyroid syndrome, neural tube defects, broncho-pulmonary dysplasia, necrotizing enterocolitis, patent ductus arteriosus, and neuronal injury in hypoxic–ischemic encephalopathy [28,29].

The exposure of TEs from donor blood has been the subject of many scientific investigations focused on specific elements, such as lead, mercury, and Cd. This exposure is exceptionally high in vulnerable groups such as neonates, preterm infants, pregnant women, and others requiring multiple blood transfusions [10,11,32]. Up to the authors’ knowledge, this is the first report of As and Se exposure in neonates via different blood product transfusions. Therefore, the aims of the study were to quantify their levels in various blood products: plasma, platelets, packed red blood cells (pRBCs), and whole blood (WB) that were used in neonatal blood transfusion alongside an estimate of dose per transfusion. In addition, database mining and molecular docking analysis were performed to explore potential detoxification strategies.

## 2. Materials and Methods

### 2.1. Sample Collection

The blood product samples came from leftover units of various blood products transfused into neonates. A total of 120 samples were obtained, with 30 samples of each type of blood product (plasma, platelets, pRBCs, and WB). Before analysis, the samples were placed in trace-mineral-free vacutainers (Becton Dickenson Co., Franklin Lakes, NJ, USA) and kept at −20 °C. All precautions were taken to minimize the risk of contamination during sample collection, aliquoting, storage, and transport to exclude any possibility of contamination.

The used blood units were prepared in the Suez Canal University Hospital blood bank by using the following method: The whole blood from the donor was collected in a triple-blood donation bag (cat# 811-5303, JMS, Hayward, CA 94541, USA) with an anticoagulant-preservative of citrate-phosphate-dextrose-adenine (CPDA-1). The primary bag contains 70 mL of CPDA-1, while the donated whole blood was 450 mL (±10%). The WB unit was loaded in the blood component centrifuge’s swinging bucket apparatus (Sorvall™ RC 128 P+, Thermo Scientific, Waltham, MA, USA), ensuring the opposing unit was balanced. The first stage separates RBC from the platelet-rich plasma (PRP) using light spin at 4 °C. At the end of the centrifuge cycle, the unit was removed from the centrifuge and placed into a plasma expressor. A hemostat was applied to the third bag tube, so the plasma flowed into only one bag. The tube was sealed and detached to separate the PRP bag with an empty satellite bag from the RBC bag. The second stage is to separate the platelets from the plasma. The PRP bag was placed in a balanced load into the blood component centrifuge’s swinging bucket apparatus and spun at a hard-spin setting. Once the centrifuge cycle had stopped, the bag was removed and placed into the plasma expressor. The temporary closure was removed, allowing the plasma to drain into an empty satellite bag, and the hemostat was applied when the approximate target platelet concentrate volume, usually 45 to 70 mL, was achieved. The tubing sealer was used to seal and detach the platelet and plasma components [33].

### 2.2. ICP-MS Analysis

Plasma, platelet, and WB samples directly underwent sample dilution before the subsequent ICP-MS analysis, while pRBCs were previously digested by mixing 400 μL of pRBCs with 800 μL of pure nitric acid (RPE analytical grade 69.5%, Carlo Erba Reagents, Cornaredo, Italy) in 15 mL capped polypropylene conical tubes (Cellstar^®^ 188271, Greiner Bio-One, Kremsmünster, Austria) at 70 °C for 1 h. This digestion significantly reduced matrix effects without bringing any other bias to the quantification. Blank mineralization samples (using ultrapure water instead of pRBCs) were systematically tested for experimental contamination. After homogenization, samples were 50-fold diluted in a 1.54% *v*/*v* nitric acid (RPE analytical grade 69.5%, Carlo Erba Reagents) solution in ultrapure water (Purelab Flex, Veolia Water, Paris, France) containing 0.1% *v*/*v* Triton^TM^ X-100 (Sigma-Aldrich, St. Louis, MO, USA) and 0.2% *v*/*v* butan-1-ol (VWR Chemicals, Radnor, PA, USA) [34].

The analysis was conducted on a THERMO ICAP^TM^ Qc ICP-MS (Thermo Scientific, Waltham, MA, USA) using helium as a collision gas to deal with polyatomic interferences. The basic operating conditions were as follows: RF Power 1550W, main argon flow 14 L/min, auxiliary argon flow 0.8 L/min, nebulizer flow 1.0 L/min, and helium flow in the collision cell 4.50 mL/min. The instrument was tuned daily for optimization. Quantification parameters are summarized in Table 1.

### 2.3. Quality Control

The quality of elemental analysis was monitored daily by measuring concentrations of reference materials used as quality controls at the beginning, periodically during the analysis sequence, and at the end ClinChek^®^ controls (RECIPE Chemicals, Munich, Germany) were used.

### 2.4. Estimation of Dose per Transfusion

The dose per transfusion (the metal exposure from each transfusion) was calculated as follows: volume transfused (L) X metal concentration in blood unit (μg/L)/weight (kg) = μg/kg/day.

### 2.5. In Silico Analysis

**Figure 1:**
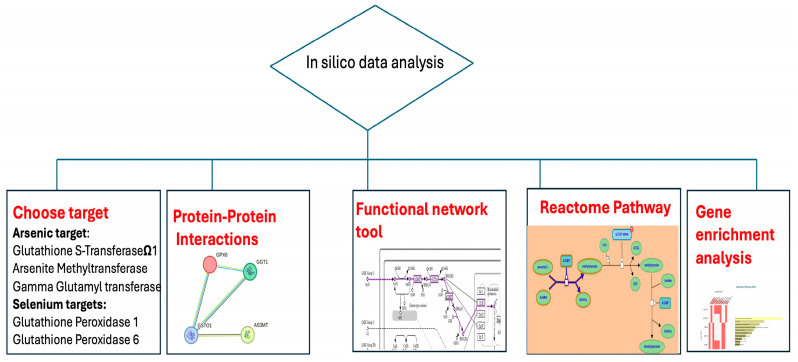
Flow chart of in silico analysis.

The workflow of the in silico analysis conducted in this study is summarized in the flowchart (Figure 1). Briefly, proteins associated with As and Se metabolism were selected based on their known biological relevance. These included Glutathione S-Transferase Omega 1 (GSTO1), Arsenite Methyltransferase (AS3MT), and Gamma Glutamyl Transferase (GGT) for As, as well as Glutathione Peroxidase 1 (GPX1) and Glutathione Peroxidase 6 (GPX6) for Se.

#### 2.5.1. Protein–Protein Interaction and Functional Enrichment Analyses

To explore potential functional interactions, a protein–protein interaction (PPI) network was constructed using the STRING database (https://string-db.org). Functional enrichment analysis was conducted using the KEGG pathway database (https://www.genome.jp/kegg/, accessed on 17 July 2025), focusing on reactive oxygen species (ROS)-related signaling. Additional pathway mapping and gene ontology (GO) annotation were performed using the Reactome Pathway Database (https://reactome.org/, accessed on 17 July 2025), DAVID (https://david.ncifcrf.gov/), and Enrichr (https://maayanlab.cloud/Enrichr/, accessed on 17 July 2025). These integrative analyses helped identify critical molecular mechanisms underlying As and Se.

#### 2.5.2. Ligand Preparation and Molecular Docking

To investigate molecular interactions at the receptor level, molecular docking studies were conducted using the crystal structure of Arsenite Methyltransferase (AS3MT) (PDB ID: 6CX6), obtained from the Protein Data Bank. The co-crystallized ligand, S-adenosylhomocysteine (ASH), was used as a reference to validate docking accuracy and compare binding affinities. The ligands selected for docking included Glutathione (GSH), S-adenosylmethionine (SAM), Folic acid, Monomethylarsonous acid (MMA III) and Resveratrol).

Ligand structures were retrieved from the PubChem database and energy-minimized. UCSF Chimera was used for receptor and ligand preparation, including removal of water molecules, addition of hydrogen atoms, and selection of the binding site based on the ASH ligand. Molecular Operating Environment (MOE) 2008 software was employed for docking simulations. The docking site was defined to encompass the ASH binding pocket to ensure a reliable comparison.

### 2.6. Statistical Analysis

Windows operating system was used for data entry, and the statistical package for social sciences (SPSS version 22) was used for data analysis. Descriptive statistics: Median, mean, and interquartile ranges (IQR) were calculated for the detectable values of As and Se concentrations to determine their distributions in different blood products. The Kruskal–Wallis (KW) test was used to compare levels of tested elements in blood products that were not normally distributed. Significance was considered at *p* ≤ 0.05.

## 3. Results

### 3.1. Quality Control

The method used in this study met appropriate laboratory quality criteria, including quality control monitoring. The analytical results of certified materials of whole blood and serum are shown in Table 2.

### 3.2. Metal Concentrations in Donor Blood Units

Metal concentrations in blood units were analyzed for 120 samples of 4 blood products (30 samples per each type of blood product). Out of 120 samples, the 2 investigated elements were detected in all samples. The As level in various blood units, arranged in descending order, is as follows: pRBCs, WBs, followed by platelets and plasma in the final position (Table 3). There are 24 donors (20%) who have As levels more than 1 μg/L. The maximum As level was observed with a concentration of 3.3 μg/L. Meanwhile, the Se level in various blood units, arranged in ascending order, is as follows: plasma, platelets, WBs, and pRBCs (Table 3). About 74% (89 donors) had Se levels below 100 μg/L. Moreover, 73 donors (60%) had Se levels less than 80 μg/L. The minimum Se concentration in the present study was 26 μg/L.

### 3.3. Dose per Transfusion

The transfusion volume was variable, ranging from 20 to 100 mL. The median doses of As and Se per transfusion of all blood product units were 0.015 and 1.4 µg/kg/day in neonates with an average weight of 1.2 kg. The median dose per transfusion and IQR dose per transfusion of different blood product units are shown in Table 4.

### 3.4. Molecular Docking Analysis

**Figure 2:**
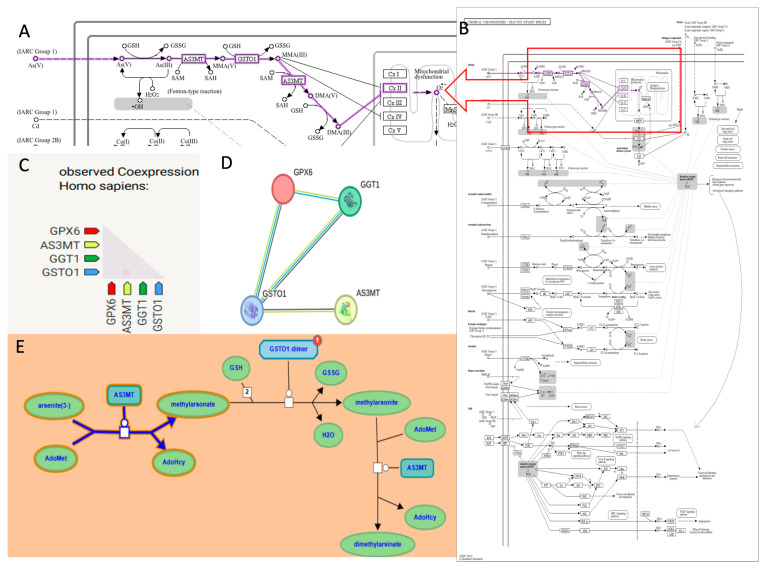
Interaction analysis for the oxidative stress pathway. (**A**,**B**) KEGG pathway of the involvement of AS3MT in arsenic biotransformation and the ROS pathway. The KEGG pathway highlights the detoxification of arsenic through methylation by AS3MT and reduction by GSTO1. (**C**,**D**) Co-expression analysis of GPX6, AS3MT, GGT1, and GSTO1 in Homo sapiens, indicating coordinated expression among these genes. STRING protein–protein interaction network showing functional associations between GPX6, AS3MT, GGT1, and GSTO1. (**E**) Reactome visualization of arsenic metabolism depicting the role of AS3MT and GSTO1 in methylation and reduction processes using substrates like GSH and intermediates such as MMA and DMA.

To better define the molecular context of As metabolism, we performed pathway and enrichment analyses of AS3MT and GSTO1 together with oxidative stress–related genes. Figure 2 integrates KEGG, STRING, and Reactome data, showing the involvement of AS3MT in As methylation and GSTO1 in reduction reactions, both using glutathione (GSH) as a key cofactor. Co-expression analysis highlighted functional associations with GPX6 and GGT1, supporting a coordinated role of these enzymes in detoxification and antioxidant defense. STRING network analysis confirmed strong protein–protein associations among AS3MT, GSTO1, GPX6, and GGT1, while the Reactome pathway representation further illustrated the sequential methylation and reduction steps leading to monomethylarsonic acid (MMA) and dimethylarsinic acid (DMA).

**Figure 3:**
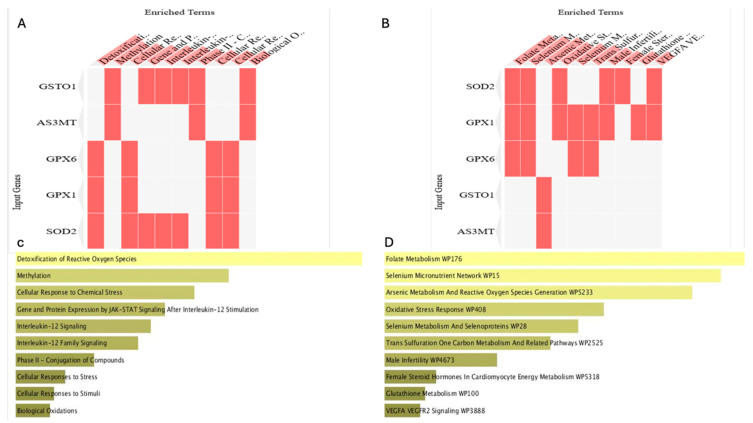
Functional enrichment analysis of genes involved in arsenic and selenium metabolism. (**A**,**B**) Enrichment analysis heatmaps of genes (AS3MT, GSTO1, GPX6, GPX1, SOD2) showing biological processes and pathways related to oxidative stress, methylation, and detoxification. Subfigure (**A**) is derived from Reactome Pathway 2024. Subfigure (**B**) is derived from WikiPathways 2024. (**C**,**D**) Enrichr pathway analysis indicating in subfigure (**C**) key roles in ROS detoxification, JAK-STAT signaling, and interleukin-mediated responses, derived from Reactome Pathway 2024. Subfigure (**D**) shows major enrichment in folate metabolism, selenium network, arsenic ROS generation, and glutathione metabolism pathways, derived from WikiPathways 2024. Analysis performed using Enrichr.

Figure 3 summarizes functional enrichment analysis of these genes. Heatmaps derived from Reactome (Figure 3A) and WikiPathways (Figure 3B) revealed enrichment in biological processes, including detoxification of reactive oxygen species, methylation, and cellular responses to chemical stress. Enrichr pathway analysis (Figure 3C,D) highlighted specific involvement in folate metabolism, Se micronutrient network, As metabolism, ROS generation, and glutathione metabolism. These findings indicate that AS3MT and GSTO1 operate within a broader oxidative stress–responsive network that integrates methylation, redox regulation, and immune-related signaling.

Figure 2 and Figure 3 provide functional evidence that AS3MT and GSTO1 are central players in As detoxification and oxidative stress regulation, which justified their further investigation by molecular docking analysis.

Table 5 shows that the molecular docking studies provided detailed insights into the interaction between Arsenite Methyltransferase (AS3MT, PDB ID: 6CX6) and selected ligands relevant to As metabolism and potential mitigation. The co-crystallized ligand S-adenosylhomocysteine (SAH) formed four hydrogen bonds with key residues ASP115, ASN173, and GLU152, along with hydrophobic interactions involving PHE150, ILE151, and VAL175, establishing the reference for docking accuracy. Among the tested compounds, folic acid exhibited the highest number of hydrogen bonds (n = 6), interacting with residues ASN173, ASP89, ARG96, and GLU152, and forming hydrophobic contacts with LEU52, ILE131, and CYS186. S-adenosylmethionine (SAM) and glutathione (GSH) each showed stable binding through four hydrogen bonds, primarily involving ASP115, ASN173, and ARG96, with favorable lipophilic interactions at LEU140, VAL137, and CYS54. The toxic metabolite monomethylarsonous acid (MMA III) formed four hydrogen bonds with ASN173, CYS92, ASP97, and ARG96, indicating strong and potentially inhibitory binding at the enzyme’s active site. Resveratrol, despite forming only three hydrogen bonds, engaged several hydrophobic residues, including PHE31 and CYS136, suggesting potential regulatory binding.

**Figure 4:**
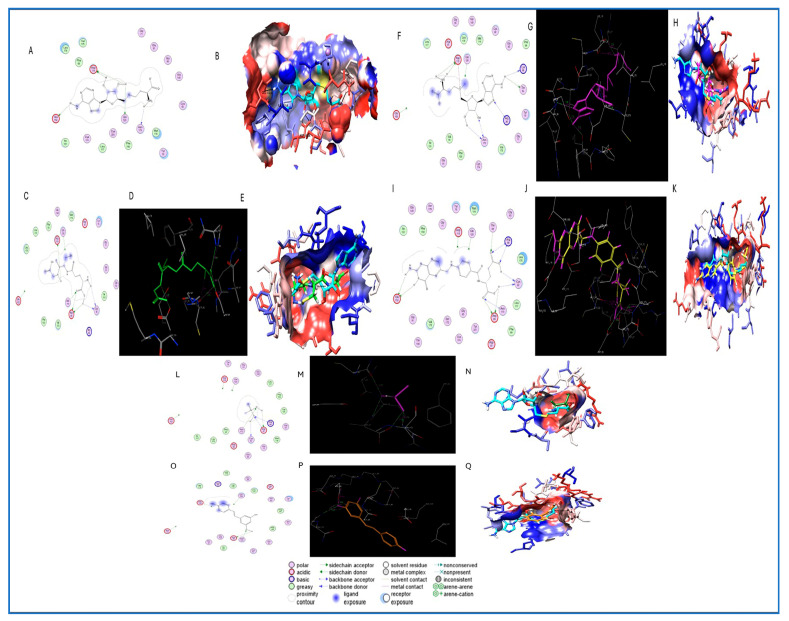
Molecular docking and interaction analysis of selected ligands with Arsenite Methyltransferase (AS3MT, PDB ID: 6CX6). (**A**–**Q**) Panels show 2D and 3D interaction maps generated using MOE and UCSF Chimera for each ligand: (**A**,**B**) SAH (co-crystallized ligand), (**C**,**D**,**E**) Glutathione (GSH), (**F**,**G**,**H**) S-adenosylmethionine (SAM), (**I**,**J**,**K**) Folic acid, (**L**,**M**,**N**) Monomethylarsonous acid (MMA III), and (**O**,**P**,**Q**) Resveratrol. Two-dimensional interaction diagrams (left) depict hydrogen bonding, donor/acceptor interactions, and residue properties. Three-dimensional surface views (middle) illustrate ligand orientation in the binding pocket, while atomic interaction details (right) highlight specific amino acid contacts.

As shown in Figure 4, molecular docking analysis using MOE revealed distinct interaction profiles between AS3MT (PDB ID: 6CX6) and six ligands involved in As metabolism or known for their protective antioxidant effects. The reference ligand S-adenosylhomocysteine (SAH) exhibited four hydrogen bonds with key residues, including ASP115, ASN173, and GLU152, confirming the active site for docking. Glutathione formed hydrogen bonds with ASP115, ASN173, CYS92, and ASP89, indicating stable binding consistent with its role in As detoxification. SAM, folic acid, and monomethylarsonous acid (MMA III) displayed multi-point interactions with residues such as ARG96, CYS92, THR94, and ASP89, reflecting strong affinity and biological relevance in methylation processes. Resveratrol, a known antioxidant, showed favorable binding with three hydrogen bonds and lipophilic contacts involving PHE31, VAL60, and LEU140. UCSF Chimera visualizations confirmed the ligands’ orientation within the AS3MT pocket, highlighting overlap with the SAH binding site and interactions with conserved active-site residues.

## 4. Discussion 

The current work is the first, to the best of our knowledge, to examine As and Se in different blood products used in blood transfusion using ICP-MS. Arsenic was detected in all blood product units investigated in this study. According to the As profile Agency for Toxic Substances and Disease Registry, the typical As values in non-exposed individuals are less than 1 μg/L [35]. There are 24 donors in the present study who have As levels more than 1 μg/L, which indicates that 20% of donors are categorized as the exposed group to As.

The two maximum As levels were observed with concentrations of 3.3 and 3 μg/L, respectively. This observation highlights the significance of careful donor selection and/or further toxicological screening for harmful TEs prior to newborn blood transfusion. According to prior studies, choosing young donors (less than 22–23 years old) for neonatal transfusions is a suggested approach to lower the possibility of negative health outcomes [36,37]. This suggestion is inconsistent with the results of the present study, in which the donor with the maximum As level was 20 years old. This indicates that this criterion of donor age could not be applied to the current study. The As-exposed donors could be accounted for by other donor variations such as smoking [38], residence in a region with high-As drinking water [39], and/or occupational exposure [40,41]. Thus, implementing donor screening questionnaires focused on smoking, living location, and occupational history could enhance the safety of blood products by donor selection.

The reviewed evidence demonstrates that supplementing storage media with antioxidants like uric acid, ascorbic acid, glutathione, and metabolites such as L-carnitine effectively counters the storage lesion by enhancing redox metabolism and reducing hemolysis [42]. This additive could be strategically applied to blood units from donors with high As or low Se levels. The safety to match units from optimal donors, aligning with the emerging principles of precision transfusion medicine [43]. This principle means moving beyond just matching ABO/Rh types to considering the unique biological characteristics of both the donor and the recipient to optimize transfusion outcomes. A donor with an ideal metabolic profile could be flagged as a “premium donor,” and their blood could be preferentially allocated to the most vulnerable patients, like neonates.

As a limitation, the present study did not measure the different As species as percentages of total As. Since it is well known that the As toxicity ultimately depends on its chemical forms. Therefore, As speciation is an important issue due to the vast differences in toxicity between species like inorganic arsenic (As^III^/As^V^) and arsenobetaine (AsB) [44]. Although it has been reported that high-performance liquid chromatography coupled with inductively coupled plasma mass spectrometry (HPLC-ICP-MS) with dynamic reaction cell has been used to separate and quantify seven As compounds (arsenobetaine, arsenocholine, Trimethylarsine oxide (TMAO), As^V^, As^III^, monomethylarsonate, and dimethylarsinate) [45]. The development of the hydride-generation cryotrapping Atomic Absorption Spectrometry (HG-CT-AAS) technique is also used for the analysis of major As human metabolites, including dimethylmonothioarsinic acid, in biological samples. The HG-CT-AAS was made to improve performance and lower detection limits [46]. But the accurate speciation remains a significant analytical challenge due to issues like low concentrations, instability of species, and matrix effects in human samples [47,48].

The highest As content in the present study was in pRBCs in comparison to the other blood products with statistical significance. This could be due to the RBCs taking up As after absorption and entering the bloodstream [12]. The highest As dose per transfusion was noticed in WB units; this could be explained by the high volume needed in case of WB transfusion [11].

It was reported that early-life exposure to As is associated with deficits in intelligence and memory. These effects may occur at low concentrations of exposure, and some neurocognitive consequences may manifest only later in life [42]. All blood units in the present study had detectable As concentrations. The Joint Food and Agriculture Organization of the United Nations (FAO)/WHO Expert Committee on Food Additives Committee declared that the previously established provisional tolerable weekly intake (PTWI) (15 μg/kg, equivalent to 2.1 μg/kg/day) of As was no longer appropriate. Therefore, this PTWI was withdrawn by this committee. No new reference dose (RfD) could be established [49,50]. There is no accepted daily dose for As because any detectable level of As is toxic [3]. We should take care that any amount of As during blood transfusion will be added to As that the newborn has received from the mother through the umbilical cord [51]. An additional risk is expected if those neonates have medical conditions that will require multiple blood transfusions.

According to the current study, neonates should be given extreme caution while making transfusion-related decisions. This is in agreement with a previous study that contrasted the outcomes of school-age children who were managed using the restricted transfusion guideline [1] to those who had inferior neurocognitive development as neonates who received numerous blood transfusions. Since it is well recognized that transfusions include some risk and it is still unclear when the advantages outweigh the concerns, this restricted transfusion guideline seems prudent and safe for neonates [52]. Otherwise, if transfusion is carried out without screening for As and other toxic TEs, donor blood may be harmful to such a vulnerable group of patients.

The results of the present study showed a wider Se range than that detected in the previous study (55–117.4 μg/L) [53]. Another study reported that the appropriate range of selenium content in human blood is 80–140 μg/L [54,55]. Meanwhile, the mean Se levels, as a comparison between the present and previous studies, 79.7 μg/L [53] and 85.6 μg/L, [56] were close. The difference in results could be accounted for if the studied populations were different.

In the present study, 74% (89 donors) had Se levels below 100 μg/L. Previous studies stated that cases with Se below 100 μg/L is a level that has been associated with sub-optimal activity of the antioxidant enzyme glutathione peroxidase (GPx) [56]. In addition, there are 73 donors (60%) with Se levels below the accepted minimum Se level (80 μg/L), which could put those neonates at risk of Se deficiency. This is consistent with Khatami et al., who revealed that blood exchange transfusion caused a 28% decrease in the blood Se level because the blood donors had lower blood Se levels than the neonates [57]. This is also consistent with McDonald and his co-workers, who displayed that transfusion of pRBCs with poor Se content can dilute Se levels and compromise glutathione peroxidase antioxidant activity and thereby allow lipid peroxidation [58].

According to a prior report, Se bulk in the body was in the tissue pool with fractional uptake of Se by RBC [59]. It has been reported that the most sensitive indicator of Se intake is RBC Se, followed by plasma Se [60]. This is consistent with the results of the present study, as the highest content was in pRBCs. Through estimation of Se loading per transfusion, it was found that the WB and pRBCs are the sources with higher Se content compared with other blood products owing to the high volume required per transfusion in the case of WB transfusion and the high Se content uptake by RBCs in the case of pRBCs. Previous studies suggested that only 2–3 mcg/kg/day can prevent further decreases in Se level in the blood of preterm infants and that higher supplementation of 7 mcg/kg/day is necessary to reach the level of healthy infants at term [61]. With the application of this suggestion in the present study, WB could provide the neonate with this suggested daily level due to transfusion.

Our molecular docking analysis revealed that key methyl donors—folic acid, S-adenosylmethionine (SAM), and glutathione (GSH)—bind stably to arsenite methyltransferase (AS3MT), interacting with conserved residues such as ASN173, ASP115, and CYS92. These interactions suggest potential modulation of AS3MT activity, a key enzyme in As biotransformation. The docking results support the hypothesis that these methyl donors and thiol-containing compounds share critical interaction sites with the co-crystallized ligand S-adenosylhomocysteine (SAH), notably ASN173 and ASP115, which are essential for enzymatic methylation of As. These findings align with previous studies demonstrating that folate and SAM supplementation enhance As methylation efficiency and reduce the accumulation of toxic intermediates like monomethylarsonous acid (MMA III) and dimethylarsinic acid (DMA V), thereby lowering arsenic-induced genotoxicity and oxidative stress in exposed populations. Gamble et al. showed that folate supplementation significantly improved As methylation capacity and reduced blood As levels in adults exposed to contaminated water [62]. This was further supported by a trial in Bangladeshi children where folic acid and vitamin B12 supplementation increased urinary excretion of dimethylarsinic acid and reduced monomethylarsonic acid, improving detoxification [63]. Additionally, polymorphisms in the AS3MT gene influence As metabolism, with certain variants associated with higher accumulation of toxic MMA metabolites and increased susceptibility to As-induced oxidative stress [64]. This is particularly relevant in neonates, especially those receiving blood transfusions, where immature enzymatic systems and limited availability of methyl donors may hinder As detoxification. Glutathione’s strong interaction, especially with CYS92 and ASP89, corroborates its known role in conjugating As species and facilitating cellular detoxification [65]. Furthermore, resveratrol exhibited favorable binding energy and key interactions with residues such as VAL60, PHE31, and CYS136, suggesting potential allosteric modulation or antioxidant protection. This is consistent with evidence demonstrating resveratrol’s ability to mitigate As-induced oxidative damage by restoring redox balance and upregulating endogenous antioxidant enzymes [66,67,68].

## 5. Conclusions

Collectively, these results highlight that neonates are exposed to As and Se via different blood product transfusions with high potential to increase As (as a toxic TE) and decrease Se (as a beneficial TE) after transfusion. This study highlights the need to select donors and screen blood units with optimal Se levels and low As for neonatal transfusions. The results indicate that methyl donors (e.g., SAM, folic acid) and antioxidants (e.g., glutathione, resveratrol) show favorable binding interactions with AS3MT, consistent with their known biochemical roles in As metabolism and redox balance. These findings are hypothesis-generating and provide a molecular basis for future experimental validation; however, direct clinical implications, such as supplementation strategies in neonates, cannot be concluded from this analysis alone.

## 6. Further Recommendations

Future in vitro and in vivo studies are needed to validate the predicted interactions of AS3MT with methyl donors and antioxidants and to assess their impact on As metabolism and detoxification. Neonatal-specific investigations, including the measurement of As metabolites in relation to folate, glutathione, and Se status, would provide important translational insights. Ultimately, if confirmed experimentally and clinically, such findings may guide the development of strategies to mitigate As toxicity in vulnerable neonatal populations.

## Figures and Tables

**Table 1:** Quantification ICP-MS parameters.

Element	Isotope	Dwell Time (s)	Sweeps per Replicate	Replicates	Internal Standard Used for Quantification	Measurement Mode	Sampling Volume (µL)	Sample Dilution	Calibration Specificity	LLOQ in Plasma, Platelets and WB (µg/L)	LLOQ in pRBCs (µg/L)
As	75	0.1	10	3	^71^Ga	KED *	100	1/50	None	0.2	0.6
Se	78	0.3	10	3	^89^Y	KED	100	1/50	None	5	15

Note: As: arsenic; Se: selenium; ^71^Ga: gallium; ^89^Y: yttrium; * KED: Kinetic Energy Discrimination, using helium in the collision cell; LLOQ: Lower limit of quantification.

**Table 2:** Summary of analytical results of certified materials of whole blood and serum.

	ClinChekwholeblood-Lot 2192				ClinChekserum-Lot2062			
	As		Se		As		Se	
	Result *	Certified ^$^	Results	Certified	Results	Certified	Results	Certified
Level 1	3.05–3.56	2.99 (2.39–3.59)	71.4–87.3	84.8 (67.8–102)	9.89–10.3	9.48 (7.58–11.4)	51.9–55.6	57.7 (46.1–69.2)
Level 2	5.57–6.16	5.37 (4.29–6.44)	139–163	163 (130–195)	19.8–20.9	19.3 (15.4–23.2)	102–104	105 (83.7–126)
Level 3	11.0–11.2	10.2 (8.14–12.2)	168–179	205 (164–245)				

Note: * Minimum and maximum concentrations of quality controls measured during the experiments, expressed in µg/L; ^$^ certified values from the material manufacturer, expressed in µg/L.

**Table 3:** Arsenic (As) and Selenium (Se) content (µg/L) in blood products transfused into neonates.

		Plasma	Platelets	pRBCs	WB	*p*
As						
	Median	0.4	0.4	0.9	0.5	0.00006 *
	IQR	0.375	0.4	0.525	0.4	
Se						
	Median	54.5	56.5	126.0	79.0	<0.00001 *
	IQR	17	17.75	25.5	15	

Note: pRBCs: packed red blood cells; WB: whole blood; IQR: interquartile range; * statistically significant determined by Kruskal–Wallis test (*p* < 0.05).

**Table 4:** Calculated arsenic (As) and selenium (Se) dose per transfusion of different blood products used for neonatal transfusion.

Dose per Transfusion		Blood Products (µg/kg/Day)		
	Plasma	Platelets	pRBCs	WB
As				
Median	0.01	0.01	0.02	0.05
IQR	0.0058	0.00625	0.0075	0.025
Se				
Median	0.9	0.9	2	7
IQR	0.283	0.296	0.425	1.25

Note: pRBCs: packed RBCs; WB: whole blood.

**Table 5:** Hydrogen bonding and hydrophobic interactions between arsenite methyltransferase (AS3MT, PDB ID: 6CX6) and selected ligands involved in arsenic metabolism.

Molecular Target and PDB Code	Compound	Hydrogen Bond Analysis			Amino Acids Involved in the Lipophilic Analysis
		N	H Bond	A	
6CX6	SAH	4	ASP115	1.8A, 1.6A	PHE 150, ILE 151, VAL175, LEU 178, METH116, CYS92
			ASN173	2.3A	
			GLU 152	2.5A	
	Glutathione conf.3	4	ASP115	2.9A	LEU52, VAL137, LEU140, LEU79, ILE113
			ASN173	3.3A	
			CYST92	2.7A	
			ASP89	3A	
	SAM conf.9	4	ASN173	2.34A	PHE31, VAL60, LEU79, LEU52, CYS54, VAL137, LEU140
			ASP115	2.25A	
			ARG96	3A	
			LYS68	3.1A	
			THR94	2.14	
	Folic acid conf.3	6	ASN173	2.7A	PHE31, LEU52, VAL60, ILE131, PHE112, LEU79, CYS186
			ASP89	3.4A	
			ARG96	2.7A	
			THR94	2.8A	
			CYST92	3A	
			GLU152	2.38A	
	MMAIII conf.4	4	ASN173	2.26A	PHE31, VAL60, CYS54, CYS136, VAL137
			CYS92	2.52A	
			ASP97	2.35A	
			ARG96	3.06A	
	Resveratrol conf.6	3	ASN173	2.02A	VAL 27, CYS54,PHE31, GLY55, LEU79, LEU140, CYS136
			ASP89	2.8A	

## Data Availability

The original contributions presented in this study are included in the article.
